# Does Housing Eviction Modify the Effects of Alcohol Outlet Density on Hospitalizations for Assault and Alcohol Use Disorder in Pennsylvania?

**DOI:** 10.1111/acer.70372

**Published:** 2026-07-09

**Authors:** Michelle Dougherty, Robert W. S. Coulter, Sarah L. Pedersen, Maya I. Ragavan, Sara E. Baumann, Christina Mair

**Affiliations:** ^1^ Department of Behavioral and Community Health Sciences, School of Public Health University of Pittsburgh Pittsburgh Pennsylvania USA; ^2^ Center for Social Dynamics and Community Health, School of Public Health University of Pittsburgh Pittsburgh Pennsylvania USA; ^3^ Department of Psychiatry University of Pittsburgh Pittsburgh Pennsylvania USA; ^4^ Division of General Academic Pediatrics University of Pittsburgh and UPMC Children's Hospital of Pittsburgh Pittsburgh Pennsylvania USA

**Keywords:** alcohol outlets, housing eviction, spatiotemporal analysis

## Abstract

**Background:**

Examining how the relationship between alcohol outlet density and alcohol‐related harms may vary by neighborhood‐level factors is important for informing the evaluation and implementation of environmental alcohol interventions and identifying other community conditions that can be modified to reduce alcohol‐related harms and inequities. This study aims to extend prior research by testing whether housing eviction—a cause of housing insecurity and neighborhood destabilization—modifies the impact of alcohol outlet density on hospitalizations for assault and alcohol use disorder (AUD) in Pennsylvania ZIP codes.

**Methods:**

We used Bayesian hierarchical space–time misalignment models to examine the associations between inpatient assault and AUD hospitalizations and housing eviction filing rates, alcohol outlet density, and the proportion of outlets selling alcohol for off‐premise consumption at the ZIP code level in Pennsylvania 2018–2022 (*n* = 7257 space–time units), reporting relative rates (RR) and 95% credible intervals (CIs). We tested whether eviction filing rates modified the associations between outlet density/the proportion of off‐premise outlets and hospitalizations for AUD and assault.

**Results:**

In main effects models, a one percent increase in the eviction filing rate was associated with a 1.2% increase in AUD hospitalizations (95% CI: 1.01, 1.015) and a 1.9% increase in assault hospitalizations (95% CI: 1.002, 1.038). The addition of one outlet per square mile was associated with a 0.3% increase in AUD hospitalizations (95% CI: 1.002, 1.004) and a 0.6% increase in assault hospitalizations (95% CI: 1.003, 1.008). In interaction models, eviction filing rates strengthened the positive associations between outlet density and hospitalizations for both assault and AUD.

**Conclusions:**

Reducing housing evictions may mitigate the impact of alcohol outlet density on AUD and assault. Future research, policy, and practice should explore opportunities for jointly addressing the alcohol environment and neighborhood housing conditions.

## Graphical abstract

This study extends prior research by examining whether housing eviction filings (1) were associated with hospitalizations for alcohol use disorder and assault and (2) modified the associations between alcohol outlet density and these outcomes in Pennsylvania 2018–2022. We found that both alcohol outlet density and housing eviction filings had well‐supported positive associations with both outcomes and that the interaction between eviction filings and alcohol outlet density was also positive and well supported.

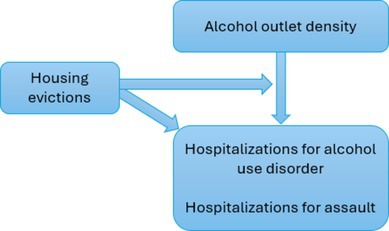

## 1 Introduction

Alcohol outlets (i.e., retail establishments that sell alcohol for consumption) are associated with a number of alcohol‐related outcomes (Babor 2023). Increased alcohol outlet density, measured as the number of outlets per capita or per area, can impact both alcohol consumption and the extent to which consumption leads to health and social problems (Campbell et al. 2009). Based on availability theory, higher alcohol outlet density in a neighborhood may increase the convenience of obtaining alcohol, in turn increasing both the volume and frequency of alcohol consumption, which can lead to alcohol use disorder (AUD) (Caetano et al. 1997; Heather and Stockwell 2004). According to niche theory, increased on‐premise alcohol outlet density can increase interpersonal violence by facilitating interactions among those consuming alcohol and by concentrating those with a propensity for violence at certain establishments (Gruenewald 2007). Bars can create environments with relaxed social controls that increase the risk of interpersonal conflict, especially if they promote heavy drinking and have poor place management (e.g., overcrowding) (Pridemore and Grubesic 2011). Overall, while many studies have linked alcohol outlet densities to increased violence, findings on the relationship between outlet density and AUD have been more mixed (Babor 2023).

Understanding whether and how alcohol outlet density interacts with other area‐level demographic and place‐based factors can help clarify the associations between outlet density and alcohol‐related outcomes. Several California‐based studies found that the association between bar density and assault was stronger in ZIP codes with lower household income/higher poverty, higher population density, a higher proportion of residents with minoritized racial and ethnic identities, and greater residential instability (i.e., a greater proportion of renters and housing turnover) (Gruenewald et al. 2006; Mair et al. 2013). Pridemore and Grubesic (2012) similarly found that the association between alcohol outlet density and assault was greater in Cincinnati, Ohio block groups with a higher proportion of rental households, household with incomes below the poverty level, and female‐headed households with children. Other studies have examined whether associations between alcohol outlet density and AUD varied by aggregate income‐level and neighborhood‐level drinking norms, but did not find support for these interactions (Ahern et al. 2015; Mair et al. 2020). Further understanding how alcohol outlet density interacts with other demographic and place‐based factors is important for informing strategies to mitigate the impacts of outlets, as well as evaluations of their effectiveness (Mair et al. 2022).

Extending this prior work to consider variation in alcohol outlet density impacts by neighborhood housing contexts is important, especially in the U.S., as these contexts are tied to a legacy of structural racism (Fullilove 2016). Although housing is a key social determinant of health, few studies have examined how housing insecurity is associated with alcohol‐related outcomes (Austin et al. 2021). Housing eviction—instances in which landlords force renters to move—is an important form of housing insecurity to examine as it is both modifiable (e.g., through eviction moratoria and diversion programs) and prevalent, impacting nearly 7% of rental households in the U.S. per year (Desmond 2016; Gromis et al. 2022). Eviction‐related experiences include both forced removal from one's home and living under the threat of eviction, an often prolonged period of stress that persists as tenants anticipate an eviction filing and court ruling (Garboden and Rosen 2019). Although eviction often occurs in the context of poverty and nonpayment of rent, it is a distinct determinant of poor health outcomes with unique geographical patterning driven by specific landlord practices (Decker 2021; Gomory 2021; Leung et al. 2021; Rutan and Desmond 2021).

Housing eviction may affect alcohol‐related outcomes in several ways. At a neighborhood level, displacement and community uprooting due to eviction can disrupt social ties, decreasing social connectedness and solidarity (i.e., social cohesion) and the reciprocal flow of resources that imbue collective power (i.e., social capital), potentially leading to more crime as a result of less social accountability and bystander intervention (Schwartz et al. 2024). A high neighborhood‐level eviction rate could increase both the overall salience of eviction threat (e.g., witnessing a neighbor being forced to leave, knowing that one's landlord is poised to use eviction as a property management strategy) and the perception that one's neighborhood is unfairly targeted due to racial stigma, increasing both psychosocial and identity‐based stressors, which may increase alcohol consumption and risk of AUD (Arcaya et al. 2014; Keyes et al. 2012; Schwartz et al. 2024).

Eviction may also interact with alcohol outlet density to exacerbate alcohol‐related outcomes, particularly assault and AUD. Eviction often occurs as part of extractive landlord management practices that also include property neglect and weak tenant screening, creating physical and social conditions that make crimes more likely to occur (Gomory and Desmond 2023). These poor landlord management strategies also include the use of eviction as a rent collection strategy, exacerbating housing cost burden and limiting tenants' control over their living conditions (Garboden and Rosen 2019). As noted above, eviction fosters environments where there is increased stress, and less social cohesion and social capital. In this context, alcohol outlets could more easily lead to increased alcohol use disorder (e.g., by making it easier to drink as a means of coping with stress) and assault (e.g., by further concentrating individuals who may be more likely to engage in crime and limiting the extent to which others can move away from crime hotspots). Increased alcohol outlet density could also create conditions (e.g., permissive drinking norms, more alcohol availability) that increase the extent to which eviction leads to increased alcohol consumption, increasing the risk of alcohol‐related harms (Ahern et al. 2008; Paschall et al. 2012).

In summary, while previous studies have shown that the associations between alcohol outlet density and negative alcohol‐related outcomes vary by neighborhood contextual factors, such as area‐level poverty, none have previously examined whether these associations vary based on area‐level eviction rates. This is an important gap to fill as eviction is a policy‐sensitive form of housing insecurity that impacts a substantial number of renters in the U.S. The aims of this paper therefore are to (1) examine the independent effects of outlet density, the proportion of outlets that are off‐premise, and eviction filings on hospitalizations for AUD and assault and (2) assess whether eviction filings modify associations between outlet density/the proportion of outlets off‐premise and hospitalizations for AUD and assault.

## 2 Methods

We used Bayesian space–time misalignment models to examine the effects of housing eviction, alcohol outlet density, and the proportion of outlets that are off premise on inpatient hospitalizations for assault and AUD at the ZIP code level across the state of Pennsylvania 2018–2022, adjusting for community sociodemographic characteristics. We also tested whether eviction filing rates modified the associations between outlet indicators and each outcome of interest. The institutional review board at the University of Pittsburgh reviewed this study and determined that it was exempt.

### 2.1 Data Sources and Measures

We linked all data for this study at the ZIP code level by year. ZIP codes correspond to post office delivery areas and can include point‐based areas, such as post office boxes. We spatially joined ZIP codes to create contiguous areas, resulting in year‐specific shapefiles with 7257 space–time units 2018–2022.

We obtained inpatient hospital discharge data for all hospitals and freestanding ambulatory centers in PA (excluding Veteran's Affairs and other federally run hospitals) (Pennsylvania Health Care Cost Containment Council 2024). These data include information on each patient's ZIP code, principal diagnosis codes, and up to 17 secondary diagnosis codes. We used ICD‐10 codes to identify hospitalizations for AUD and assault, creating aggregate counts of each of these hospitalization types by ZIP code (See File S1 for ICD‐10 codes used to identify hospitalizations for each of these conditions). Hospitalizations were counted if the associated ICD‐10 code was included either as a primary or secondary diagnosis code. Of 7,829,185 inpatient hospitalizations in PA 2018–2022, we excluded 5.0% because the residential ZIP code was invalid or the patient was living outside of PA.

We obtained a list of all active liquor licenses from the Pennsylvania Liquor Control Board (PLCB) in 2025 (PLCB+ Online Regulatory System 2025). We restricted this dataset to establishments regularly selling alcohol for on‐ or off‐premise consumption, including restaurants, beer distributors, breweries/brewery pubs, limited wineries and distilleries, taverns, and eating places (establishments such as corner stores and delis selling beer and food for primarily off‐premise consumption) based on the descriptions of these establishment types provided by the PLCB. Entities that did not regularly sell alcohol for consumption (e.g., importers, manufacturers, special occasion permits) were excluded. Since this dataset contained multiple rows per license number documenting the history of each license, we cleaned these data to deduplicate addresses, creating a listing of unique alcohol outlet addresses. The locations of state‐run liquor stores were obtained separately from the PLCB annual reports (Pennsylvania Liquor Control Board 2019). We geocoded addresses of alcohol outlets using Geocodio (Geocodio 2025). Of 13,280 addresses, 3.9% could not be geocoded or were outside of PA, leaving the addresses of 12,765 outlets for analysis. We identified outlets selling alcohol for primarily off‐premise consumption, including state‐run liquor stores, beer distributors and eating places, in line with prior analyses of alcohol outlets in PA (Grubesic et al. 2013). The PLCB does not grant different license types for bars vs. restaurants, thus we did not differentiate these outlet types in our analyses. Prior research has revealed that outlets that close tend to be replaced by other outlets; thus, while specific outlets may vary from year‐to‐year, the geographic distribution is likely stable over time (Gruenewald et al. 2022). Using QGIS (version 3.34.11), we generated an aggregate count of all outlets and divided this by the area (in miles^2^) of each ZIP code to calculate overall outlet density (Open Source Geospatial Foundation Project 2025). Using the area as the denominator for alcohol outlet density is an appropriate approach for measuring spatial access that yields easily interpretable estimates (Sacks et al. 2020). We also calculated the proportion of all alcohol outlets per ZIP code selling alcohol for off‐premise consumption.

We obtained eviction filing court records from the Magisterial District Judge System from 2018 to 2022 for all of PA, with the exception of Philadelphia, which we obtained from Philadelphia Legal Assistance (Philadelphia Legal Assistance 2024; The Unified Judicial System of Pennsylvania 2025). We reviewed eviction records and excluded cases in which the ZIP code of the defendant (i.e., tenant) was missing, invalid or outside of PA, and limited cases to residential eviction by searching defendant names for key terms associated with businesses (e.g., corporation LLC) in line with best practices (Desmond et al. 2018). Of 460,669 cases, 99.2% (*n* = 456,948) were included for analysis across 2018–2022. We included eviction records regardless of what the case disposition (i.e., court ruling) was as these fields were often incomplete. Eviction filings are often used as proxy for forced moves due to eviction and are indicative of eviction threat, an important health exposure onto itself (Garboden and Rosen 2019; Gromis et al. 2022). We calculated eviction filing rates by aggregating counts of eviction filings per ZIP code per year and divided this by the number of rental households in that ZIP code, obtained from the U.S. census.

For socioeconomic and demographic characteristics, we used 5‐Year American Community Survey (ACS) tract‐level estimates for each year of analysis and extrapolated estimates to the ZIP code level. To do this, we assigned census tract‐level estimates to the census blocks within each tract, then spatially linked ZIP codes to census blocks to calculate population weights (i.e., the proportion of each block's population that was within each ZIP code), which we then applied to the tract‐level estimates associated with each block, summing these within ZIP codes (similar to a weighted average). Socioeconomic and demographic variables included the percent of total population across age groups (percent < 18, 18–24, 25–44, 45–64, and ≥ 65), percent male, race and ethnicity distributions (percent Non‐Hispanic or Latino White, Non‐Hispanic or Latino Black, Hispanic or Latino, and all other races and ethnicities), percent of population age ≥ 15 married, percent of total workforce unemployed, percent of total population with incomes below the federal poverty level, median household income per $10,000, percent of those age ≥ 25 with a college degree, and the percent insured. We categorized population density into equal quintiles across PA (corresponding to 0–55.9, 60.0–140.1, 140.2–407.0, 407.1–1742.0 and > 1742.90 people per mile^2^) and divided these values by 100 to ensure that credible intervals would be wide enough to interpret. We also used indicators of the local housing environment from the ACS, including the percent of households experiencing rent burden (paying > 30% of income on rent) and the percentage of vacant housing units.

To capture healthcare utilization and availability, we calculated the number of hospitalizations per 100 people in each ZIP code. We also obtained the locations of all hospitals in PA and locations of facilities providing substance use and/or mental health (SUMH) treatment (Pennsylvania Spatial Data Access 2025; Substance Abuse and Mental Health Services Administration 2025). Using QGIS (version 3.34.11), we aggregated the number of hospitals and the number of facilities providing SUMH treatment by ZIP code by year (Open Source Geospatial Foundation Project 2025). Since these health care resources were sparsely distributed (only 13% of ZIP codes had a hospital and 25% had a SUMH treatment facility), we created binary indicators capturing if there was one or more hospital or SUMH treatment facility per ZIP code (two binary indicators, one for each).

Eleven ZIP codes had populations less than five. For these ZIP codes, we changed the ZIP code population count to five and replaced the values of other variables with the year‐specific statewide mean to ensure there was a non‐zero risk of hospitalizations in every ZIP code. Finally, we included year‐specific dummy variables in our analyses to control for differences across years not explained by other covariates and likely due to temporal trends.

### 2.2 Analysis

We first calculated descriptive statistics, including the mean and distribution of each variable. Data preparation and descriptive analyses were conducted in Stata (version 18.0) (StataCorp 2023).

Next, we examined the relationships between eviction filings, overall alcohol outlets density, the proportion of outlets off‐premise, and hospitalizations for AUD and assault by ZIP code‐year using Bayesian hierarchical space–time misalignment models. Consistent with prior research (Caetano et al. 2021; Mair et al. 2013), we include overall outlet density and the proportion of outlets off‐premise together in our models to examine the influence of overall alcohol availability vs. the influence of outlet composition. Since ZIP code boundaries shift by year, we accounted for spatial misalignment between years by including separate conditional autoregressive (CAR) random spatial and nonspatial effects for each space–time unit in our models (Zhu et al. 2013). We assigned uninformed priors for all fixed and random effects. We used Poisson regression for all models as all outcomes were counts.

$$Y_{i,t}\;|\;\mu_{i,t}\sim \text{Poisson}\;\left(E_{i,t}\;\exp.\left(\mu_{i,t}\right)\right)$$

In this equation, *Y*_*i,t*_ is the count of hospitalizations for each ZIP code *i* in year *t*. *E*_*i,t*_ is the expected count of hospitalizations under the assumption that these hospitalizations are distributed directly proportional to total ZIP code population. The relative rate for each space–time unit is represented by exp. (*μ*_*i,t*_). The log relative rate *μ*_*i,t*_ is modeled as:

$$\mu_{i,t=}\;{\mathrm{\beta X}}_{i,t}+\phi_{i,t}+\theta_{i,t}$$

In this equation, *β* is the vector of coefficient estimates for each fixed effect, while *X*_*i,t*_ is the matrix of fixed effects. ϕ_*i,t*_ is the vector of CAR spatial random effects and θ_*i,t*_ is the vector of nonspatial random effects.

We used the R‐INLA package to fit the models using integrated nested Laplace approximation (INLA), a deterministic approach used as an accurate alternative to Markov Chain Monte Carlo (MCMC) approaches (Carroll et al. 2015; Held et al. 2010; Lindgren and Rue 2015). Since these models account for changes in ZIP code boundaries across years, they do not allow for a longitudinal assessment of changes within ZIP codes over time. For each outcome (hospitalizations for assault and AUD), we tested a main effects model and an interaction model with two interaction terms: eviction filings interacted with outlet density and eviction filings interacted with the proportion of off‐premise outlets (four total models). To build our models, we assessed each fixed effect as it was added and removed fixed effects that were not well‐supported, assessing whether removal affected other fixed effects. We included a consistent set of fixed effects across models to facilitate comparison.

Models provide posterior distributions for each fixed effect. We calculated the relative rate of each fixed effect using the medians of these posterior distributions. To determine if fixed effects were well supported, we used 95% credible intervals, which identify the range in which 95% of the posterior distribution falls for each fixed effect. All Bayesian space–time models were run in R version 4.4.2 (R Core Team 2024).

## 3 Results

From 2018 to 2022 there were 7,438,646 inpatient hospitalizations in PA. Of these, 5.82% (*n* = 358,367) had primary or secondary diagnoses for AUD and 0.05% (*n* = 3394) had primary or secondary diagnoses for assault. As shown in Table 1, an average of 0.48% (SD: 0.46%) of residents were hospitalized for AUD and 0.01% (SD: 0.02%) were hospitalized for assault per ZIP code per year. On average, 3.88% (SD: 4.64%) of renter households per ZIP code per year received eviction filings. There was an average of 2.13 alcohol outlets per square mile, although there was substantial variation (SD: 15.58). Twelve percent of these outlets were off‐premise on average.

**TABLE 1:** Characteristics of Pennsylvania ZIP codes by year 2018–2022.

	2018–2022 (*N* = 7257 ZIP code‐years)				
	Mean	Median	SD	Min	Max
Total population, *N*	8865.58	3830.62	11,469.20	3.98	71,271.25
Alcohol use disorder hospitalizations, *N*	49.38	16.00	83.15	0.00	888.00
Alcohol use disorder hospitalizations, % (of total population)	0.48	0.41	0.46	0.00	15.05
Assault hospitalizations, *N*	0.47	0.00	1.83	0.00	37.00
Assault hospitalizations, % (of total population)	0.01	0.00	0.02	0.00	0.70
Eviction filings, % (per rental households)	3.88	2.62	4.64	0.00	65.91
Male, %	50.01	49.84	2.58	38.53	82.25
Age					
Under 18, %	20.19	20.19	3.68	0.57	35.23
18–24, %	8.25	7.36	5.23	2.56	89.53
25–44, %	22.97	22.17	4.39	4.31	64.99
45–64, %	28.72	29.12	3.60	2.64	60.84
65 and over, %	19.87	20.11	3.91	1.51	40.95
Race/Ethnicity					
Hispanic or Latino, %	4.15	2.06	6.70	0.00	69.47
Non‐Hispanic or Latino Black, %	4.84	1.21	10.76	0.00	89.07
Non‐Hispanic or Latino White, %	87.06	93.05	15.84	4.19	100.00
All Other Races & Ethnicities^a^, %	3.95	2.73	3.54	0.00	27.23
Married, %	25.52	26.78	5.01	0.37	37.16
Population with college degree, %	15.91	13.76	7.24	4.67	61.17
Unemployed, %	3.09	2.87	1.25	0.37	11.24
Income below poverty level, %	10.77	9.89	5.78	1.02	50.78
Median income, per $10 k	6.73	6.14	2.24	1.81	22.95
Rent burden, % of rental households	35.82	35.27	10.07	0.87	87.61
Vacant housing, % of housing units	20.33	17.36	11.22	2.43	81.98
Insured, %	92.19	93.92	5.89	34.08	99.62
All hospitalizations, per 100 people	2.73	2.69	1.36	0.00	31.69
Presence of hospital^b^	932	12.84			
Presence of substance use and mental health treatment facility^b^	1810	24.94			
Alcohol outlet density, per mi^2^	2.13	0.13	15.58	0.00	910.43
Proportion of alcohol outlets off premise	0.12	0.00	0.18	0.00	1.00

Abbreviation: SD = standard deviation.

^a^ Includes those identifying as Non‐Hispanic/Latino American Indian and Alaska Native, Asian, Native Hawaiian and Other Pacific Islander, and multiple races.

^b^ Indicates number and percent of ZIP codes with at least one hospital or SUMH treatment facility.

The results of Bayesian space–time misalignment models for AUD and assault hospitalizations in Pennsylvania for 2018–2022 are shown in Table 2. In the main effects model for AUD (Model 1a), a one percent increase in the eviction filing rate was associated with a 1.2% increase in risk for AUD hospitalizations (95% CI: 1.01, 1.015). Both alcohol outlet density (RR: 1.003, 95% CI: 1.002, 1.004) and the proportion of outlets off premise (RR: 1.142, 95% CI: 1.085, 1.203) were associated with increased hospitalizations for AUD. Year‐specific fixed effects show that the relative rate of AUD hospitalizations increased over time. A number of other fixed effects had a well‐supported association with increased AUD hospitalizations: a lower percentage of Hispanic or Latino residents, a lower percentage of residents with other minoritized racial identities (excluding those with Black identities), a higher percentage of individuals ages 45–64, a lower percentage of those who were married, an increased percentage of those unemployed, higher poverty, lower median household income, an increased percentage of households with rent burden, increased percentage of vacant housing units, as well as an increased percent of those insured. Additionally, an increased overall hospitalization rate, having a hospital, and having a SUMH facility in a ZIP code were all independently associated with increased AUD hospitalizations.

**TABLE 2:** Relative rates and 95% credible intervals of hospitalization for AUD and assault estimated from spatial misalignment models for 2018–2022.

	AUD		Assault	
	Model 1a RR (95% CI)	Model 1b RR (95% CI)	Model 2a RR (95% CI)	Model 2b RR (95% CI)
Eviction filing rate, % per rental households	1.012 (1.010, 1.015)^a^	1.011 (1.008, 1.013)^a^	1.019 (1.002, 1.038)^a^	1.016 (0.995, 1.038)
Outlet density, per mi^2^	1.003 (1.002, 1.004)^a^	1.002 (1.001, 1.003)^a^	1.006 (1.003, 1.008)^a^	1.005 (1.002, 1.008)^a^
Proportion of outlets off premise	1.142 (1.085, 1.203)^a^	1.140 (1.068, 1.217)^a^	1.111 (0.734, 1.680)	1.149 (0.683, 1.933)
Eviction filings × outlet density		1.004 (1.003, 1.005)^a^		1.008 (1.002, 1.013)^a^
Eviction filings × proportion off premise		1.000 (0.963, 1.039)		0.967 (0.725, 1.287)
Year (ref: 2018)				
2019	1.029 (1.005, 1.054)^a^	1.028 (1.004, 1.052)^a^	1.188 (0.918, 1.536)	1.188 (0.919, 1.536)
2020	1.039 (1.013, 1.065)^a^	1.037 (1.010, 1.063)^a^	0.989 (0.746, 1.311)	0.984 (0.742, 1.307)
2021	1.097 (1.068, 1.126)^a^	1.100 (1.071, 1.130)^a^	1.126 (0.859, 1.478)	1.138 (0.868, 1.493)
2022	1.108 (1.076, 1.142)^a^	1.111 (1.078, 1.145)^a^	0.739 (0.542, 1.009)	0.750 (0.549, 1.024)
Population density, people per sq. mile/100 (ref: Quintile 1)				
Quintile 2	0.991 (0.955, 1.029)	0.996 (0.960, 1.035)	0.741 (0.569, 0.966)^a^	0.756 (0.580, 0.985)^a^
Quintile 3	1.053 (1.009, 1.100)^a^	1.064 (1.019, 1.112)^a^	0.806 (0.604, 1.075)	0.839 (0.629, 1.12)
Quintile 4	1.024 (0.974, 1.077)	1.041 (0.990, 1.094)	0.698 (0.504, 0.966)^a^	0.739 (0.533, 1.026)
Quintile 5	0.991 (0.933, 1.052)	1.004 (0.946, 1.065)	0.759 (0.523, 1.101)	0.797 (0.549, 1.157)
Race/Ethnicity, % (ref: Non‐Hispanic or Latino White)				
Hispanic or Latino	0.996 (0.994, 0.998)^a^	0.996 (0.994, 0.998)^a^	0.973 (0.961, 0.986)^a^	0.970 (0.958, 0.983)^a^
Non‐Hispanic or Latino Black	0.998 (0.997, 1.000)	0.998 (0.997, 1.000)	0.999 (0.992, 1.007)	0.999 (0.991, 1.006)
All Other Races & Ethnicities	0.995 (0.991, 0.999)^a^	0.995 (0.991, 0.999)^a^	0.999 (0.977, 1.022)	0.998 (0.976, 1.021)
Age, % (ref: 45–64)				
Under 18, %	0.979 (0.974, 0.985)^a^	0.980 (0.975, 0.986)^a^	1.015 (0.980, 1.051)	1.018 (0.983, 1.054)
18–24, %	0.966 (0.962, 0.970)^a^	0.967 (0.962, 0.970)^a^	0.963 (0.939, 0.988)^a^	0.963 (0.938, 0.987)^a^
24–44, %	0.986 (0.980, 0.991)^a^	0.986 (0.981, 0.992)^a^	1.009 (0.979, 1.040)	1.008 (0.978, 1.039)
65+, %	0.978 (0.972, 0.984)^a^	0.979 (0.973, 0.985)^a^	1.001 (0.964, 1.039)	1.000 (0.964, 1.039)
Married, %	0.963 (0.956, 0.969)^a^	0.962 (0.956, 0.969)^a^	0.928 (0.892, 0.966)^a^	0.928 (0.892, 0.965)^a^
Unemployed, %	1.004 (0.993, 1.015)	1.004 (0.993, 1.015)	1.033 (0.968, 1.102)	1.030 (0.966, 1.100)
Income below poverty level, %	1.006 (1.003, 1.010)^a^	1.005 (1.002, 1.009)^a^	1.017 (0.997, 1.037)	1.015 (0.996, 1.036)
Median income, per $10 k	0.975 (0.966, 0.985)^a^	0.972 (0.963, 0.982)^a^	0.950 (0.890, 1.016)	0.939 (0.878, 1.004)
Rent burden, % of rental households	0.998 (0.997, 0.999)^a^	0.998 (0.997, 1.000)	1.000 (0.990, 1.009)	1.000 (0.991, 1.010)
Vacant housing, % of housing units	1.003 (1.001, 1.005)^a^	1.002 (0.999, 1.004)	1.003 (0.991, 1.015)	1.000 (0.988, 1.012)
Insured, %	1.015 (1.013, 1.018)^a^	1.016 (1.013, 1.018)^a^	0.997 (0.981, 1.013)	0.997 (0.981, 1.012)
All hospitalizations, per 100 people	1.28 (1.267, 1.294)^a^	1.275 (1.262, 1.289)^a^	1.212 (1.132, 1.297)^a^	1.194 (1.111, 1.281)^a^
Hospital in ZIP code	1.045 (1.023, 1.068)^a^	1.049 (1.026, 1.071)^a^	1.143 (1.015, 1.287)^a^	1.149 (1.021, 1.294)^a^
Substance Use and Mental Health Treatment Facility in ZIP code	1.040 (1.019, 1.06)^a^	1.041 (1.021, 1.062)^a^	1.057 (0.943, 1.185)	1.058 (0.943, 1.186)
Spatial random effect (SD CAR process)	0.310 (0.306, 0.315)	0.305 (0.301, 0.310)	1.621 (1.604, 1.639)	1.614 (1.597, 1.632)
ZIP code‐level random effects (SD)	0.126 (0.106, 0.148)	0.130 (0.111, 0.151)	0.034 (0.015, 0.096)	0.034 (0.015, 0.093)
Spatial‐to‐total random variability ratio	0.858 (0.814, 0.896)	0.846 (0.803, 0.883)	0.999 (0.997, 0.999)	0.999 (0.997, 0.999)

*Note:* All Other Races & Ethnicities includes those identifying as Non‐Hispanic/Latino American Indian and Alaska Native, Asian, Native Hawaiian and Other Pacific Islander, and multiple races.

Abbreviations: AUD = alcohol use disorder, CAR = conditional autoregressive, CI = credible interval, IPV = intimate partner violence, RR = relative rate, SD = standard deviation.

^a^ Finding is well supported by the data as indicated by the credible interval excluding 1.

In Model 1b, we tested interaction terms between eviction filings and outlet density, as well as the proportion of off‐premise outlets. While the interaction between eviction filings and the proportion of off‐premise outlets was not well supported, the interaction between eviction filings and outlet density was positive and well‐supported (RR: 1.004, 95% C: 1.003, 1.005). This means that as the eviction filing rate increases, the association between outlet density and AUD hospitalizations strengthens. Also, as outlet density increases, the association between eviction filings and AUD hospitalizations increases.

In our main effect model for assault hospitalizations (Model 2a), a 1% increase in the eviction filing rate was associated with a 1.9% increase in assault hospitalizations (95% CI: 1.002, 1.038). The association between outlet density and assault hospitalizations was also positive and well‐supported (RR: 1.006, 95% CI: 1.003, 1.008), while the association between the proportion of outlets off premise and assault hospitalizations was not. Similar to AUD hospitalizations, a higher proportion of residents who were Hispanic or Latino and a percentage of those who were married were both protective against assault hospitalizations. An increase in the overall hospitalization rate and having a hospital in a ZIP code were associated with increased assault hospitalizations. In the interaction model for assault hospitalizations (Model 2b), the interaction between eviction filings and outlet density was positive and well‐supported (RR: 1.008, 95% CIL 1.002, 1.013), while the interaction between eviction filings and the proportion of outlets off premise was not.

Across models, the spatial‐to‐random variability ratio indicated that the CAR spatial random effect explained 84.6%–99.9% of the overall error variance, indicating a very high degree of spatial dependence in these models and highlighting the importance of correcting for spatial random effects. Not accounting for these effects can result in increased type I error. The zip code‐level random effects were also greater than zero across all models, indicating the importance of also controlling for differences across block groups that are not spatially correlated.

## 4 Discussion

There is a substantial body of literature showing that increased alcohol outlet density is associated with negative alcohol‐related outcomes, including AUD and assault (Babor 2023). This study makes a novel contribution to the extant research by showing that eviction filing rates were directly associated with increased hospitalizations for AUD and assault and strengthened the well‐supported positive associations between alcohol outlet density and these outcomes. Results from this study comport with prior research showing that the impacts of alcohol outlets on assault are higher in areas with lower household income/poverty, greater residential instability, higher population density, and a higher proportion of residents with minoritized racial and ethnic identities, renters, and female‐headed households with children (Gruenewald et al. 2006; Mair et al. 2013; Pridemore and Grubesic 2012). These economic and demographic characteristics tested in prior studies are closely related to housing eviction; eviction is more common in urban areas, is closely associated with poverty and rental instability, and disproportionately burdens Black women with children (Desmond and Gershenson 2017).

Findings from this study underscore the importance of intervening to reduce alcohol‐related impacts in communities dually burdened by eviction and high alcohol outlet density. Both of these exposures tend to disproportionately burden communities with lower incomes and—in the U.S.—communities with a high proportion of residents with minoritized racial and ethnic identities (Berke et al. 2010; Desmond and Gershenson 2017; Han 2023; Rutan and Desmond 2021). Areas that were “redlined” in the 1930s—a racist practice used by the U.S. Federal Housing Authority to designate lower income areas with residents with minoritized racial and ethnic identities as high risk for mortgage lending—have higher present‐day alcohol outlet density (Lee et al. 2020). Evictions are also part of the U.S.'s legacy of housing discrimination. Landlords have significant latitude over when they decide to pursue eviction, making it a complex, interpersonal process influenced by gender and racial dynamics; for example, ethnographic work has revealed that individuals who are White and/or male are often able to more successfully negotiate with landlords when behind on rent (Desmond 2016). On a structural level, evictions occur alongside inequitable urban renewal practices and gentrification, continually destabilizing racially marginalized communities (Fullilove 2016). Addressing the disproportionate burden of alcohol‐related impacts on lower income populations and those with minoritized racial and ethnic identities requires continued attention to inequitable risk exposures (i.e., eviction and alcohol outlet density) and the way in which historical and contemporary power structures (e.g., racism) shape environments to influence alcohol‐related outcomes.

This study has several limitations. We used inpatient hospitalizations to measure AUD and assault, which likely only capture the most severe cases of these conditions, missing those that are treated in other care settings (e.g., emergency, Veteran Affairs where hospitalization rates for these conditions may be higher), untreated (e.g., because of lack of access to health care or because they result in death before care can be accessed), or not recorded (e.g., due to differences in provider coding). Using secondary diagnosis codes (as opposed to only primary diagnosis codes) to capture hospitalizations for the outcomes of interest has the benefit of capturing more cases; however, there may be variability by condition, provider type, and patient demographic in the extent to which these codes are applied when not the primary reason for hospitalization. Eviction filing records likely underestimate the impacts of eviction as they do not capture informal evictions (e.g., when tenants leave after being threatened with a filing but before they receive an eviction notice); however, self‐reported eviction data are only available at the metropolitan level in selected areas (Gromis et al. 2022). Despite these limitations, both sets of data are consistently collected across time and space at the ZIP code level, enabling spatial analysis at a finer spatial resolution than at the county or state level.

There is also uncertainty surrounding the categorization of off‐premise outlets in our analysis for several reasons: (1) “eating places,” which we categorized as off‐premise and typically include stores (e.g., pizza places) where food and alcohol is sold for off‐premise consumption, are licensed to serve up to 30 patrons on‐premise, meaning that they can sometimes serve as on‐premise outlets (Pennsylvania Liquor Control Enforcement 2025); (2) restaurants and bars, which we did not categorize as off‐premise sites in our analysis, can sell up to 192 fluid ounces of beer to‐go in PA, meaning that they sometimes function as off‐premise outlets; (3) loitering by patrons in the areas around off‐premise outlets (e.g., parking lots, alleyways) can mean these outlets sometimes function similar to on‐premise locations, a limitation we partially address by looking at overall outlet density (Ghanem et al. 2020); and (4) bars and restaurants were closed in PA from March 17, 2020 to June 5, 2020 and may have operated as off‐premise rather than on‐premise outlets during this time (Alcohol Policy Information System 2022). Additionally, because PA does not grant different license types to bars vs. restaurants (Pennsylvania Liquor Control Enforcement 2025), we were unable to separate these two outlet types in our analyses, a notable limitation as other studies have found that associations with assault vary across these outlet types (Gruenewald et al. 2014; Snowden and Pridemore 2013).

Results from this study should be interpreted at the appropriate ecological level. Because our analysis was conducted at the ZIP code level, it would not be appropriate to make inferences about individual‐level eviction experiences, exposure to alcohol outlets and risk of hospitalization for alcohol‐related harms. However, using an ecological approach to examine these exposures and outcomes is important as there are many potential mechanisms linking them that may operate at an aggregate level (Morrison et al. 2016; Schwartz et al. 2024). It is also important not to interpret our findings as causal. Although we controlled for an array of fixed effects, there is likely unmeasured confounding. In particular, our analysis period includes the COVID‐19 pandemic, which began on March 11, 2020 and persisted through 2022. We included fixed effects for each year, but because we use ZIP code‐years as the unit of analysis we are unable to control for the specific time period in which the COVID‐19 pandemic occurred. During this period, policy changes reduced eviction (e.g., through moratoria), limited the sale of alcohol (e.g., through the closure of retail establishments), and may have jointly influenced these exposures and alcohol‐related outcomes. For example, increased COVID‐19 cases may have been positively related to eviction risk and/or alcohol‐related outcomes, while the provision of financial aid to individuals and businesses (e.g., emergency rental relief, assistance for small businesses, unemployment assistance) may have reduced both eviction risk and/or alcohol‐related outcomes. It is challenging to control for these and other potentially countervailing influences given their breadth and a lack of consistently collected, geographically well‐resolved data measuring their impacts. Extending these analyses to future years would be useful for examining whether the associations found in this study persist.

There is also the possibility of reverse causation, especially with respect to eviction and alcohol‐related hospitalizations. We chose to examine eviction as an exposure because we were interested in how it potentially modified the influence of alcohol outlet density on assault and AUD. This study used ecological panel data, meaning that we were unable to assess longitudinal trends (e.g., changes within ZIP codes over time) and potential bidirectional relationships. In the future, multilevel, longitudinal data could enable the better examination of trajectories of alcohol use and related harms, including how they relate to individual‐level exposures (e.g., personal eviction risk, contact with alcohol outlets) and neighborhood‐level exposures (e.g., living in a neighborhood with a high eviction rate, high alcohol outlet density) over time.

This study has several implications for future research and interventions efforts. Eviction moratoria, rental assistance, landlord‐tenant mediation, and access to civil legal aid have all shown success in reducing eviction (Ellen et al. 2021; Kong et al. 2022; Marçal et al. 2023). These strategies may be useful for both reducing assault and AUD directly and indirectly through reducing the impacts of alcohol outlet density on these outcomes. This is important as state pre‐emption laws can curtail the ability of local government officials to reduce alcohol outlet density in the U.S. (Mosher and Treffers 2013). In PA, legislation passed in 2016 expanded the number of off‐premise outlets and legislation proposed in 2022 would likely lead to additional increases, further underscoring the need strategies for mitigating the public health impacts of alcohol outlets (Grubesic et al. 2012; Mihalek 2022; Pennsylvania Liquor Control Board 2016). Beyond reducing the number of alcohol outlets in communities, there are several other environmental alcohol interventions that have shown promise in reducing the impact of outlets on alcohol consumption and related impacts, such as limiting the hours and days of alcohol sales (Babor 2023). It would be useful to examine whether the impacts of these policies vary across different neighborhood contexts and whether they interact with eviction to reduce alcohol‐related impacts. The collection of local alcohol policy data is important for pursuing this line of research. Furthermore, future research should examine whether these policies reduce the disproportionate burden of alcohol‐related harms on individuals with minoritized identities. Appropriately powered multilevel analyses could help facilitate this.

Overall, this research speaks to the importance of multipronged approaches for reducing alcohol‐related impacts, especially among communities inequitably burdened by these harms. Many policy‐level efforts to address alcohol‐related outcomes focus on regulating the availability and pricing of alcohol as well as who can drink and in what context (National Institute on Alcohol Abuse and Alcoholism 2025). Pursuing broader social policies—such as eviction prevention policies—in conjunction with alcohol‐specific policies as part of a multisectoral “health in all policies” approach has strong promise for advancing health equity (Hall and Jacobson 2018). Identifying the right mix of policy and intervention levers for reducing alcohol‐related inequities is an important priority for future research. On a community level, it is also important to engage local residents dually burdened by eviction and high alcohol outlet density to identify intervention opportunities. Participatory, qualitative approaches, such as photovoice, which build collective awareness of how environmental exposures influence alcohol‐related outcomes and enhance collective efficacy for addressing these exposures, have strong potential (Baumann et al. 2025; Dhital et al. 2023). Future research should examine the potential mechanisms linking eviction, the alcohol environment, and alcohol‐related outcomes. Clarifying these mechanisms could help identify multipronged policy and intervention approaches for reducing alcohol‐related harms in communities experiencing a disproportionate burden of these harms.

## Author Contributions

M.D. conceptualized this study, conducted all analyses, and was responsible for writing the manuscript draft. C.M. supervised the study and acquired funding and data in collaboration with M.D. R.W.S.C., S.L.P., M.I.R., and S.E.B. provided additional oversight and reviewed and edited the manuscript.

## Funding

This research was supported in part by a grant from the National Center for Advancing Translational Sciences (UL1TR001857). Baumann is supported by the Fogarty International Center of the National Institutes of Health under award number K01TW012424. The content is solely the responsibility of the authors and does not necessarily represent the official views of the National Institutes of Health.

## Conflicts of Interest

The authors declare no conflicts of interest.

## Supporting information


**File S1:** ICD‐10 diagnosis codes used to identify alcohol‐related hospitalizations.

## Data Availability

Research data are not shared.
