# Supplementary material for: Does Housing Eviction Modify the Effects of Alcohol Outlet Density on Hospitalizations for Assault and Alcohol Use Disorder in Pennsylvania?
Source: Alcohol Clin Exp Res (Hoboken). 2026 Jul 9;50(7):e70372. doi: 10.1111/acer.70372 (PMC13347276; doi:10.1111/acer.70372)
Supplement: Supplementary file 1 — File S1: ICD‐10 diagnosis codes used to identify alcohol‐related hospitalizations. [file ACER-50-0-s001.docx]

**Supplement. ICD-10 Diagnosis Codes Used to Identify Alcohol-Related Hospitalizations**

| **Hospitalization Type** | **ICD-10 Codes** |
| --- | --- |
| Alcohol use disorder^1^ | F10* |
| Assault and homicide^2^ | X92-Y08, Y09 |

^1.^ The Centers for Disease Control (CDC) Alcohol-Related Disease Impact (ARDI) System (<https://www.cdc.gov/alcohol/ardi/alcohol-related-icd-codes.html>) defines alcohol abuse and dependence as ICD-10 codes F10.0-F10.2. In our inpatient hospital discharge data, hospitalization with F10.0-F10.2 constituted 98.3% of all hospitalizations with any F10* code (based on primary and secondary diagnoses). Other researchers (Bernstein et al., 2023; Manca & Lewsey, 2024; Riedel et al., 2024; Suen et al., 2022) have commonly use F10* (i.e., including F10.3-F10.9), including alcoholic psychosis, in their definitions of AUD, thus we also included these codes in our definition as well.

^2.^ Codes based on Centers for Disease Control (CDC) Alcohol-Related Disease Impact (ARDI) System (<https://www.cdc.gov/alcohol/ardi/alcohol-related-icd-codes.html>)

**References**

Bernstein, E. Y., Baggett, T. P., Trivedi, S., Herzig, S. J., & Anderson, T. S. (2023). Pharmacologic Treatment Initiation Among Medicare Beneficiaries Hospitalized With Alcohol Use Disorder. *Annals of Internal Medicine*, *176*(8), 1137–1139. https://doi.org/10.7326/M23-0641

Manca, F., & Lewsey, J. (2024). Previous psychiatric hospitalizations as risk factors for single and multiple future alcohol-related hospitalizations in patients with alcohol use disorders. *Addiction*, *119*(2), 291–300. https://doi.org/https://doi.org/10.1111/add.16352

Riedel, O., Braitmaier, M., Dankhoff, M., Haug, U., Klein, M., Zachariassen, W., & Hoyer, J. (2024). Alcohol use disorders after bariatric surgery: a study using linked health claims and survey data. *International Journal of Obesity*, *48*(11), 1656–1663. https://doi.org/10.1038/s41366-024-01606-3

Suen, L. W., Makam, A. N., Snyder, H. R., Repplinger, D., Kushel, M. B., Martin, M., & Nguyen, O. K. (2022). National Prevalence of Alcohol and Other Substance Use Disorders Among Emergency Department Visits and Hospitalizations: NHAMCS 2014-2018. *Journal of General Internal Medicine*, *37*(10), 2420–2428. https://doi.org/10.1007/s11606-021-07069-w
